# Complex gaze stabilization in mantis shrimp

**DOI:** 10.1098/rspb.2018.0594

**Published:** 2018-05-02

**Authors:** Ilse M. Daly, Martin J. How, Julian C. Partridge, Nicholas W. Roberts

**Affiliations:** 1 School of Biological Sciences, University of Bristol, Tyndall Avenue, Bristol BS8 1TQ, UK; 2 School of Biological Sciences and the Oceans Institute, Faculty of Science, University of Western Australia, 35 Stirling Highway, Crawley, Western Australia 6009, Australia

**Keywords:** stomatopod, gaze stabilization, optokinesis, eye movements, neural connections

## Abstract

Almost all animals, regardless of the anatomy of the eyes, require some level of gaze stabilization in order to see the world clearly and without blur. For the mantis shrimp, achieving gaze stabilization is unusually challenging as their eyes have an unprecedented scope for movement in all three rotational degrees of freedom: yaw, pitch and torsion. We demonstrate that the species *Odontodactylus scyllarus* performs stereotypical gaze stabilization in the yaw degree of rotational freedom, which is accompanied by simultaneous changes in the pitch and torsion rotation of the eye. Surprisingly, yaw gaze stabilization performance is unaffected by both the torsional pose and the rate of torsional rotation of the eye. Further to this, we show, for the first time, a lack of a torsional gaze stabilization response in the stomatopod visual system. In the light of these findings, we suggest that the neural wide-field motion detection network in the stomatopod visual system may follow a radially symmetric organization to compensate for the potentially disorientating effects of torsional eye movements, a system likely to be unique to stomatopods.

## 1. Introduction

Moving animals are confronted with a visual trade-off: their eyes are more efficient at detecting salient features of a scene and local motion cues when they are fixed relative to the outside world and yet, for many tasks, having movable eyes provides an adaptive advantage. Overcoming this problem is a visual challenge that has resulted in the evolution of systems that steady the retinal projection of the external visual scene for periods of time. This is achieved with eye movements that counter movements of the visual field.

If an animal's eyes were to stay immobile as its head or body moves, the direction of visual gaze would become displaced and the retinal image would be distorted and degraded due to motion blur. Motion blur occurs mainly due to the relatively slow response time of photoreceptors (typically greater than 20 ms in vertebrates and approx. 12–24 ms in invertebrates [1–3]). It is more difficult to detect an object, either stationary or in motion, relative to its background in a blurred image than in a spatio-temporally stabilized one [2,4]. Additionally, motion blur disrupts an animal's ability to infer information from optic flow or motion parallax [2,4]. Furthermore, without adequate visual compensation for rotational and translational movements of the body, an animal's egocentric coordinate system can become misaligned with real-world coordinates, so body posture and equilibrium may become compromised [4]. To counteract these degrading visual effects, animals make compensatory movements with their eyes, head or body depending on their individual anatomy to reduce movement of the retinal image [2]. This is known as gaze stabilization, and is common to both vertebrates and arthropods.

The main mechanisms known to control gaze stabilization are the vestibular–ocular reflex (VOR), the optokinetic response (OKR) and the optomotor response (OMR). The VOR involves slow counter rotation of the eyes that compensates for head motion and is triggered by signals from sense organs in the vestibular system such as the semicircular canal organs (e.g. humans [5–7], monkeys [8,9] and rabbits [10,11]), ampullae (e.g. fish [12,13]) or hair cells in statocysts (e.g. crabs [14] and lobsters [15]). The OKR, on the other hand, is mediated only by vision. It consists of a repetitive series of eye movements with a slow and a fast phase, known as the optokinetic nystagmus [1,2,16–19]. In the OKR, the eye typically performs a slow rotation in the same direction as the movement of the visual scene followed, at intervals, by a rapid counter rotation, which ‘flicks’ the eye back to the approximate starting position [1,16,18]. The slow phase of optokinetic nystagmus largely ‘fixes’ an image on the retina and is seen in animals both with and without a fovea or acute zone. The OMR is similar to the OKR, but involves movement of the entire body not just the eyes [16].

Gaze stabilization is thought to be important for an animal to be able to perceive motion accurately. There is an abundance of motion-sensitive interneurons in the arthropod central nervous system [20–23]. Most of these are directionally specific to some degree and while some wide-field neurons are directly involved in the gaze stabilization response, other neurons with a smaller receptive field require stabilization for optimal performance [24–26]. An example of such neurons can be found in the lobula of male fleshflies (*Sarcophaga bullata*). These retinotopic directionally sensitive neurons have a small receptive field and are linked to flight motor neurons, indicating that they may play a role in the tracking of females during sustained aerial pursuit [24]. Some directionally insensitive neurons associated with optic flow have been identified in the lobular plate of crustaceans and insects, responding to both vertical and horizontal motion [20,27,28].

**Figure 1.**
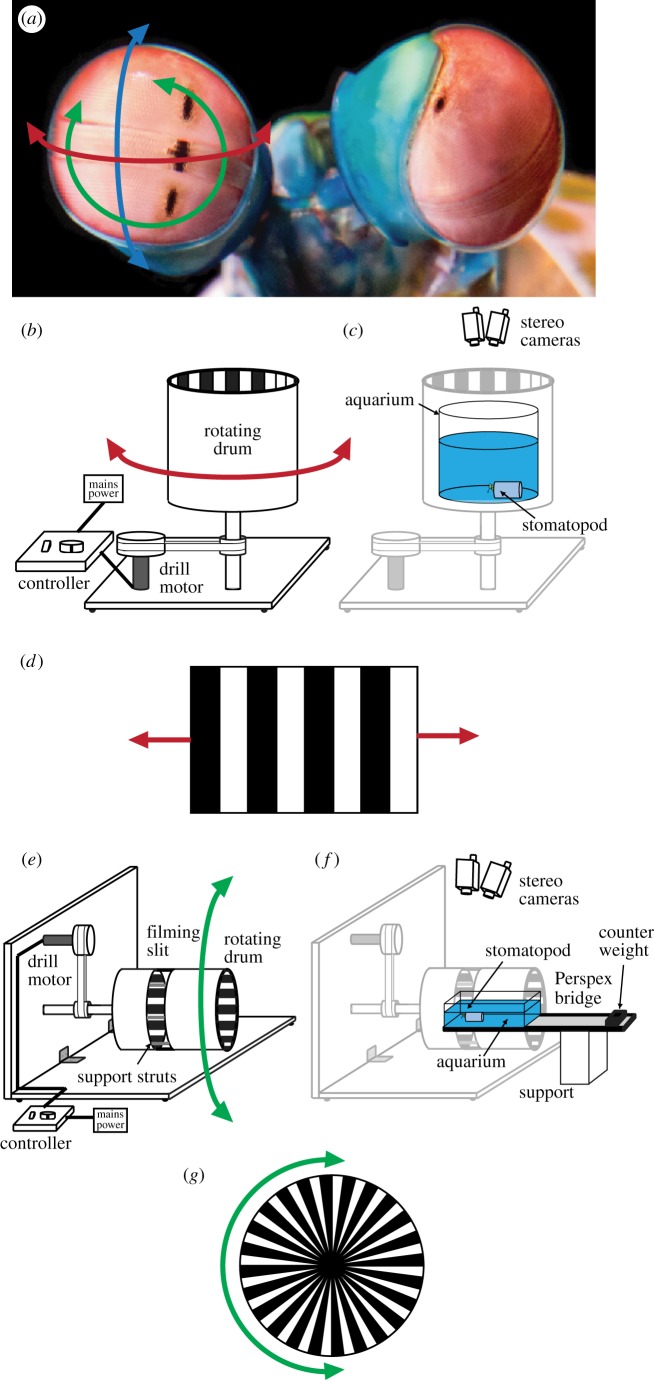
(*a*) Stomatopods can rotate their eyes in all three degrees of freedom: yaw (red; side-to-side), pitch (blue; up-down) and torsion (green; rotation about the stalk). Photo credit: Mike Bok. (*b*) The rotating drum with the black and white grating on the inner face used to elicit yaw optokinesis. (*c*) Individual stomatopods are placed in the stationary aquarium with their body concealed within an artificial ‘burrow’ and their exposed eyes, located at the centre of the drum, are filmed using stereoscopic cameras from above. (*d*) Motion of the rotating drum (*b*,*c*) creates a field of view moving in the horizontal direction. (*e*) The torsional rotating drum. As for (*a–c*), the sides of the drum were covered with a black and white grating. (*f*) Individual stomatopods are placed in the stationary counterbalanced aquarium in the middle of the drum and filmed from above through slits in the drum. (*g*) The end of the drum (left in (*d*,*e*)), directly in front of the stomatopod, was filled with a radial pattern of black and white segments, which rotated torsionally at the same rate as the drum. (Online version in colour.)

While most animals endeavour to restrict the movement of their eyes for all the reasons stated previously, stomatopod crustaceans (mantis shrimp) have unusually mobile eyes. Their compound eyes are of the apposition type, and while their eye anatomy shares many similarities with that of many other crustaceans, they show a uniquely regionalized structure. Each eye has three sections: the dorsal and ventral hemispheres, and a midband typically consisting of two or six ommatidial rows (depending on the species) bisecting the eye and anatomically separating the hemispheres [29–35]. Various adaptations to the basic crustacean photoreceptor anatomy, particular to each section of the eye, have enabled stomatopods to evolve regional specializations for 12-channel colour vision, as well as for both linear and circular polarization vision [30,31,36–40]. Stomatopod eyes also exhibit an unusually large angular range of movement in which their eyestalks move in all three degrees of rotational freedom (figure 1*a*), exceeding 90° in pitch (up-down movements), yaw (side-to-side) and torsion (rotation about the visual axis) [41,42]. Additionally, the eyes show a high degree of independence, though this depends on the visual task [43].

Despite their unique eye design, stomatopods perform stereotypical eye movements including optokinesis [18,44], tracking [45] and object-of-interest acquisition through saccades [46]. More unusual are the scanning motions of the eyes directed approximately perpendicular to the midband [44]. Because the midband consists of up to six ommatidial rows and the direction of view of ommatidia in the hemispheres is skewed, the field of view of the stomatopod eye is much reduced compared with that of many crustaceans with superficially similar apposition compound eyes. For example, the midband ommatidia typically only view a narrow 10° strip of space [31,47–49]. Consequently, scans made perpendicular to the midband will obtain sequential spectral and polarization information across a greater portion of the visual scene, rather than just a narrow strip; much like push-broom sensors used for remote sensing [50]. Despite this need for scanning, stomatopods show stereotypical gaze-stabilizing eye movements, performing yaw or pitch optokinesis in response to a horizontally or vertically displaced field of view, respectively, to stabilize the retinal image [18,43,44]. By contrast, the role of torsional eye rotation for gaze stabilization in stomatopods is much less clear [42,43].

Torsional rotation, in which the eye rotates about the long axis of the eyestalk, is an unusual movement in an animal with frontally placed eyes. Stomatopods have previously been shown to use torsional rotations to enhance their polarization vision [42]. However, the greater than 90° range of torsional rotation far exceeds the range needed for dynamic polarization vision (22.5°), suggesting that these eye rotations have additional functions. In this work, we investigate the role of torsional eye movements during gaze stabilization in the stomatopod *Odontodactylus scyllarus* and ask three questions: do the eyes rotate torsionally during optokinetic responses to a horizontally displaced field of view? Do torsional rotations affect the yaw gaze stabilization performance? Is there evidence for gaze stabilization in the torsional degree of freedom?

## 2. Material and methods

Full details of materials and methods are presented in the electronic supplementary material, S1. Yaw optokinesis was elicited by the horizontal motion of a black and white grating on the inner face of a rotating drum (figure 1*b–d*), following the method of Daly *et al*. [43]. A torsionally rotating field of view was created by turning the drum on its side such that its axis of rotation was horizontal (figure 1*e–g*). The closed end of the drum, which the animals were facing, was covered by a radial pattern of stripes so as to extend the torsionally rotating field of view frontally (figure 1*g*). The three-dimensional rotation of the eyes was recorded using two video camcorders (Panasonic HC-X900, Osaka, Japan) calibrated to form a stereoscopic pair and tracked in each frame (50 fps) using MATLAB (2015b, Mathworks, Massachusetts, USA) using the method previously described by Daly *et al*. [42]. The performance of an eye when stabilizing its gaze was quantified using the relative velocity ratio (*S*_Y_ and *S*_T_ in the yaw and torsion degrees of rotation, respectively, previously termed ‘gain’ [18]), which is the ratio between the angular velocity of the drum and the angular velocity of the eye in a particular plane of rotation. The angular velocity of the eyes is derived from the differential of the pose in each video frame, rather than manually selecting regions of the responses in order to ensure equal numbers of measurements from each eye and each individual was included in the analyses.

### (a) Statistical analyses

All statistical analyses were conducted in R v.3.0.25 [51]. The mean and standard deviation are quoted for normal distributions, and the median and 95% confidence interval (CI) for non-normal distributions of independent data. Gaze stabilization performance was analysed using a generalized linear mixed-effects model (GLMM) (R package lme4, [52]). Correlation between the three degrees of rotational freedom and between torsional rotation and yaw gaze stabilization performance was investigated using cross-correlation on the differential of the data series with respect to time in order to satisfy the stationarity assumption (i.e. that there is no overall trend in the data, such that the mean and variance do not change over time) [53] and to avoid the potential influence of high-frequency noise on the correlation calculation. Both the maximum cross-correlation coefficient between combinations of eye movements and the associated time lags were determined separately for the left and right eyes of each individual and were statistically analysed using a Wilcoxon signed-rank test to ascertain whether there was evidence of significant correlations. An additional Wilcoxon analysis was performed to determine whether the torsional pose of the eye, horizontal or vertical, had a significant effect on the median yaw gaze stabilization performance. Statistical analysis of the effect of the drum velocity on the torsional velocity of the eye also used a GLMM.

## 3. Results

### (a) Yaw optokinesis

**Figure 2.**
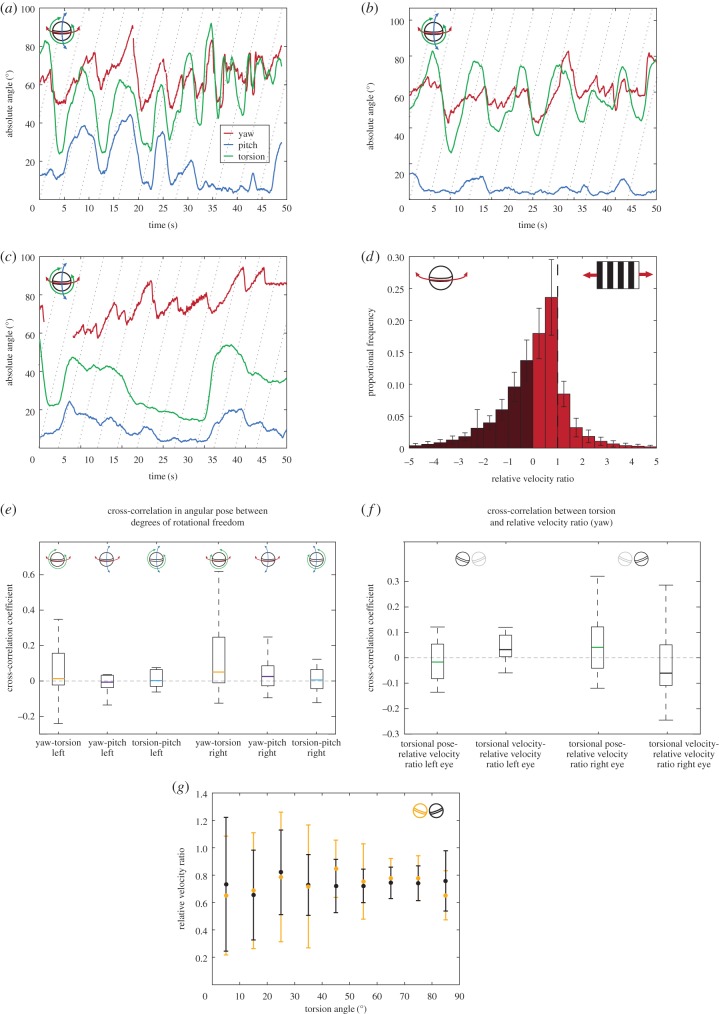
(*a–c*) The three-dimensional rotational response (yaw (red), pitch (blue) and torsion (green)) of the left eye of a single stomatopod during three separate trials in which the striped drum rotated anticlockwise in the yaw plane, producing a horizontally moving field of view. (*d*) The distribution of relative velocity ratios during the fast and slow phases of yaw optokinesis across the left and right eyes of 17 *O. scyllarus* during presentation of the drum rotating in both directions. Dashed vertical line indicates ‘perfect’, idealized gaze stabilization (*S*_Y_ = 1). *S*_Y_ > 0 when the eye is yawing in the same direction as the drum and *S*_Y_ < 0 when yawing in the opposite direction (dark red region), as occurs during fast resets (*n* = 17, error bars are standard deviation across all animals in each 0.5 interval). (*e*) Distribution of the cross-correlation coefficients between the angular pose of each of the degrees of eye rotation during yaw-plane experiments showing non-significant correlation between yaw and torsion, yaw and pitch, and torsion and pitch for the left and right eyes (*n* = 17). Horizontal dashed line indicates a cross-correlation coefficient of 0. (*f*) Boxplot of the cross-correlation coefficients between the relative velocity ratio in the yaw degree of freedom and the torsional rotation of the left and right eyes during yaw-plane experiments. Yaw gaze stabilization performance is independent of both torsional pose and velocity of torsional rotation. Horizontal dashed line indicates a cross-correlation coefficient of 0. (*g*) Median values of relative velocity ratio in 10° intervals as the left (orange) and right (black) eyes rotate torsionally from horizontal (0°) to vertical (90°) (*n* = 17, error bars are the standard deviation across all animals in each 10°interval).

Seventeen individual *O. scyllarus* performed stereotypical yaw optokinesis with slow tracking and fast reset profiles (figure 2*a*–*c*, red line) with variable kinetics (electronic supplementary material, S2), which would serve to partially stabilize their gaze to a horizontal displacement of their field of view during the tracking phase. There was no significant difference in the yaw gaze stabilization performance (*S*_Y_ in the direction of drum rotation throughout the duration of the trial) between the left and right eyes (left: *S*_Y_ = 0.74 ± 0.01 (median ± 95% CI), right: *S*_Y_ = 0.74 ± 0.01 (median ± 95% CI), GLMM, *n* = 17, *Χ*^2^ = 0.19, *p* = 0.665). Nor did the direction of rotation have a significant effect on yaw gaze stabilization performance (clockwise: *S*_Y_ = 0.74 ± 0.01 (median ± 95% CI), anticlockwise: *S*_Y_ = 0.73 ± 0.01 (median ± 95% CI), GLMM, *n* = 17, *Χ*^2^ = 2.61, *p* = 0.106; figure 2*d*). Across the whole distribution of *S*_Y_, taking the velocity of counter rotations and tracking rotations into account, the median of the relative velocity ratios is significantly greater than 0 (*S*_Y_ = 0.60 ± 0.01 (median ± 95% CI), Wilcoxon sign-ranked test, *n* = 17, *V* = 153, *p* < 0.001), indicating that the eye movements made by the stomatopods are mostly for gaze stabilization.

Despite the drum movement creating a field of view moving only in the horizontal plane, stereotypical OKR yaw rotations were accompanied by both torsion and pitch. The median torsional pose relative to the horizontal was 67.34 ± 0.12° (median ± 95% CI, *n* = 17) and the median pitch pose relative to the horizontal was 16.64 ± 0.08° (median ± 95% CI, *n* = 17). The profile of pitch and torsion rotations during yaw optokinesis are highly variable, sometimes showing apparently correlated rotation (figure 2*a*), while other times showing highly uncorrelated rotation (figure 2*b*,*c*). However, overall the median maximum cross-correlation coefficient was not significantly different from 0 for yaw and torsion rotation, yaw and pitch rotations or pitch and torsion rotation (Wilcoxon signed-rank test, *n* = 17, *V* = 117, *p* > 0.05, full statistics in electronic supplementary material, S3; figure 2*e*). These findings demonstrate that stomatopods are able to independently rotate their eyes in the three degrees of rotational freedom, but that these rotations can become coupled in some instances, such as the yaw and torsion rotation during optokinesis shown in figure 2*a*,*b*. The reason for this occasional coupling is, as yet, unclear.

Yaw gaze stabilization performance (*S*_Y_) was unaffected by the torsional pose (*θ*_T_) of the eye. There was no strong overall correlation between *S*_Y_ and *θ*_T_: the correlation coefficients between these variables for the left eye are not significantly different from 0 (left: −0.02 ± 0.08 (median ± 95% CI), Wilcoxon signed-rank test, *n* = 17, *V* = 59, *p* = 0.407; figure 2*f*) and, while the right eye correlation was significantly different from 0, the correlation coefficient indicates an extremely weak correlation (right: 0.03 ± 0.28 s (median ± 95% CI), Wilcoxon signed-rank test, *n* = 17, *V* = 122, *p* = 0.031; figure 2*g*). Further to this, the torsional pose of the eye, when divided into the categories ‘horizontal’ (0° ≤ *θ*_T_ ≤ 25°) or ‘vertical’ (65° ≤ *θ*_T_ ≤ 90°), had no significant effect on the median value of *S*_Y_ when the eye was oriented in either angular category (left horizontal: 0.65 ± 0.05 (median ± 95% CI); left vertical: 0.75 ± 0.02 (median ± 95% CI), Wilcoxon signed-rank test, *n* = 17, *V* = 70, *p* = 0.600; right horizontal: 0.72 ± 0.06 (median ± 95% CI); right vertical: 0.87 ± 0.02 (median ± 95% CI), Wilcoxon signed-rank test, *n* = 17, *V* = 73, *p* = 0.890; figure 2*g*). Similarly, there is no correlation between yaw gaze stabilization performance and the velocity of torsional rotations, with the maximum cross-correlation coefficients being not significantly different from 0 (left eye: 0.04 ± 0.13 (median ± 95% CI), Wilcoxon signed-rank test, *n* = 17, *V* = 113, *p* = 0.084; right eye: 0.04 ± 0.21 (median ± 95% CI), Wilcoxon signed-rank test, *n* = 17, *V* = 56, *p* = 0.332; figure 2*g*).

### (b) Torsional optokinesis

**Figure 3.**
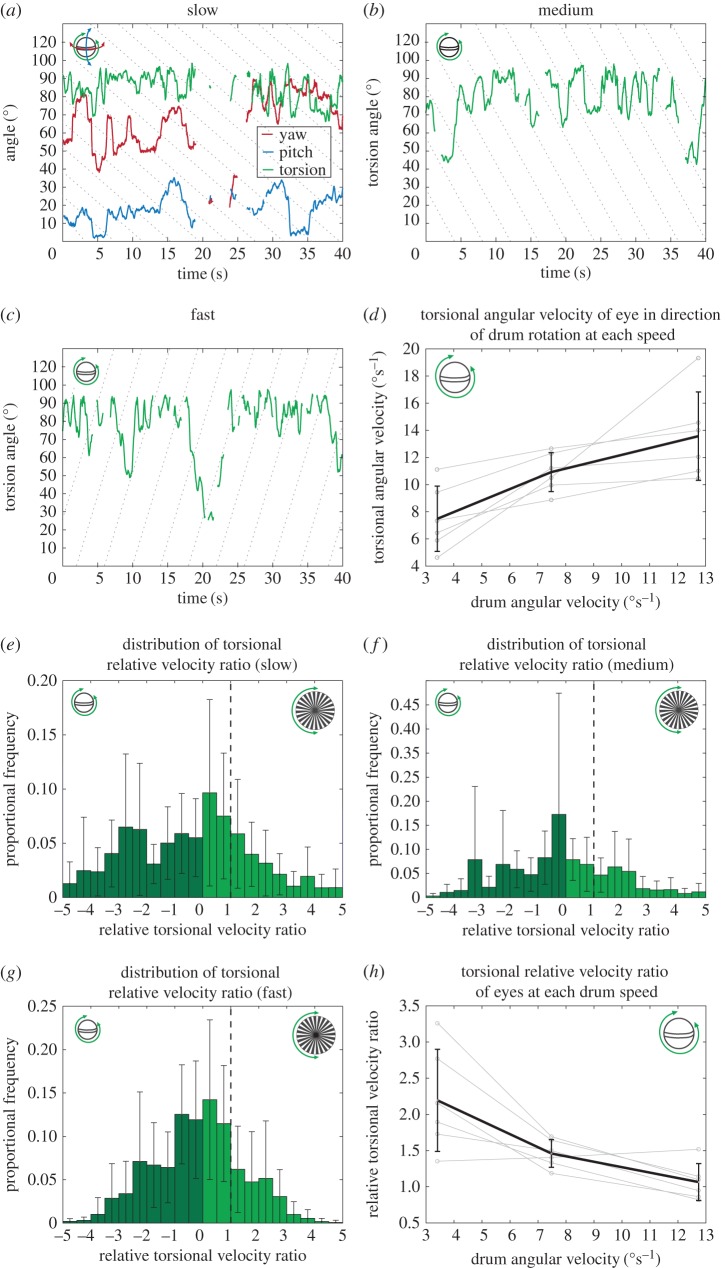
(*a*) The yaw (red), pitch (blue) and torsion (green) rotation of a single eye from an individual elicited by the torsional rotation of the drum in the clockwise direction at the slow speed setting (3.41 ± 0.04°s^−1^ (mean ± s.d.)). (*b*,*c*) Torsional rotation at the medium (7.48 ± 0.42°s^−1^; clockwise) and fast (12.74 ± 0.16°s^−1^; anticlockwise) speeds. Dotted lines (*a–c*) indicate the progress of the torsionally rotating drum, but do not necessarily represent specific stripe boundaries. Torsion of the eye does not show stereotypical optokinetic nystagmus. Missing regions are due to occlusion of the eyes by the support struts of the rotating drum. (*d*) Average angular torsional velocity of both eyes (left and right) of all six individuals (black line) increased with the angular velocity of the drum. Error bars are the standard deviation at each drum speed (*n* = 6). Also shown (grey) are the average angular torsional velocities of both eyes of each individual. (*e–g*) Distribution of relative velocity ratios during the fast and slow phases of torsional optokinesis across both eyes of six animals during clockwise and anticlockwise presentations at the (*e*) slow, (*f*) medium and (*g*) fast speed settings. The dashed line indicates ‘perfect’, idealized gaze stabilization, *S*_T_ = 1. As for figure 2*d*, counter rotation indicated by the dark green region. Error bars are the standard deviation across all animals in each 0.5 interval (*n* = 6). (*h*) Average torsional relative velocity ratios of both eyes of all six individuals (black line) at each drum speed setting all exceed *S*_T_ = 1. Error bars are the standard deviation at each drum speed (*n* = 6). Also shown (grey) are the average torsional relative velocity ratios of both eyes of each individual. While eye velocity approaches the drum velocity (*S*_T_ ≈ 1), gaze-stabilizing eye movements are expected to be slightly slower (*S*_T_) than the drum movements due to the finite response time of the neural feedback loop.

The torsional rotation of the drum elicited torsional rotation of the eyes, as well as yaw and pitch rotations (figure 3*a*). However, there was no evidence for torsional gaze stabilization as the angular torsional velocity of the eye poorly matched the angular torsional velocity of the drum at all three speed settings (slow, medium and fast, indicated by the dotted lines, figure 3*a–*c). Nor did the torsional rotation of the eye fit the bi-phasic slow/fast profile typical of optokinetic nystagmus OKR, as was observed for yaw optokinesis (figure 2*a–c*). Nevertheless, the torsional angular velocity of the drum rotation did have a significant effect on the torsional angular velocity of the eye when it was rotating in the same direction as the drum (GLMM, *n* = 6, *Χ*^2^ = 41.31, *p* < 0.001; figure 3*d*), the eyes rotating faster in torsion at higher drum speeds. It is not clear what is causing this effect and, at this stage, we cannot eliminate the possibility that the response is to a non-visual stimulus, such as noise or vibrations from the drive motor, that increased at the higher speeds.

Unlike the relative velocity ratios in the yaw degree of rotational freedom, which showed a skewed distribution (figure 2*d*), the distributions of the average torsional relative velocity ratios at each of the three speed settings follow approximately normal distributions (figure 3*e–g*). As stated previously, only torsional data in which the eye was facing forwards (*θ*_Y_ < 30°) are included in the statistical analysis. Despite the significant effect of drum angular velocity on eye angular velocity, the average torsional relative velocity ratio (*S*_T_) in the direction of the drum for each speed setting was greater than 1 (slow: *S*_T_ = 3.09 ± 4.26, medium: *S*_T_ = 2.04 ± 1.98, fast: *S*_T_ = 1.23 ± 1.07 (mean ± s.d., *n* = 6), figure 3*h*), indicating that the eyes generally rotated faster than the drum. Neither the eye, left or right (GLMM, *n* = 6, *Χ*^2^ = 2.68, *p* = 0.102), nor the direction of drum rotation, clockwise or anticlockwise (GLMM, *n* = 6, *Χ*^2^ = 0.43, *p* = 0.513), had a significant effect on the torsional relative velocity ratio. Across the whole distribution, including the velocity of counter rotational data, the torsional relative velocity ratios are not significantly different from 0 (slow: 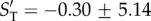 (mean ± s.d.), Wilcoxon signed-rank test, *V =* 6, *p =* 0.438; medium: 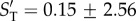, *V =* 11, *p =* 1; fast: 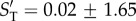 , *V =* 6, *p =* 0.400 (*n =* 6)). In other words, the eyes spend approximately as much time torsionally rotating counter to the drum direction (dark green region in figure 3*e–g*) as they do rotating in the same direction (light green region in figure 3*e–g*). This is in contrast to the eye movements in response to the yaw rotation of the drum, in which the eyes spend more time rotating in the same direction as the drum in order to stabilize their gaze as much as possible.

## 4. Discussion

When presented with a horizontally displaced field of view comprising black and white vertical stripes on a surrounding drum, *O. scyllarus* performed stereotypical yaw optokinesis in order to stabilize their gaze. The left and right eyes performed gaze stabilization equally and with no significant preference for clockwise or anticlockwise movement of the striped drum. This rotation in the yaw degree of freedom was accompanied by both pitch and torsion rotation of the eyes, despite the motion of the drum being purely in the horizontal (yaw) plane. Moreover, the yaw gaze stabilization performance was not significantly correlated with the eye's torsional or pitch pose, nor the rate of torsional rotation. A similar result was found in another stomatopod species, *Pseudosquilla ciliata* [18], suggesting that the consistency in yaw gaze stabilization performance as the eye rotates torsionally is likely to be a fundamental facet of stomatopod vision.

**Figure 4.**
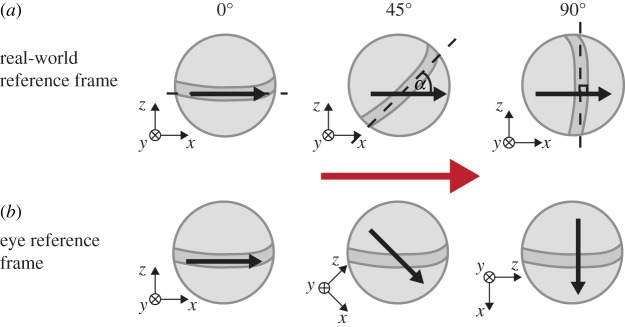
(*a*) The motion of a stimulus moving in the horizontal direction (red arrow) in the real-world coordinate system (indicated by the axes) across a stomatopod's eye depends on its torsional pose. (*b*) In its reference frame, rather than an eye torsionally rotating, it remains motionless with the midband fixed in the horizontal position, while the world rotates torsionally about the eye, as shown by the orientation of the real-world coordinate axes. In the eye's reference frame, the apparent direction of motion of the stimulus moving horizontally in the real world depends on its torsional pose. Despite the ubiquitous torsional rotations observed during yaw tracking causing a dynamic apparent direction of motion, stomatopods are able to accurately track the actual motion of a horizontally displaced field of view, showing normal optokinetic nystagmus in the yaw rotation, despite simultaneous (but uncorrelated) pitch and torsion rotations. (Online version in colour.)

The ability of stomatopods to show optokinetic stabilization in yaw, while their eyes simultaneously rotate in pitch or torsion, indicates that the neuronal network for detecting wide-field motion in the stomatopod eye must be more complex than a simple system of a Reichardt-like motion detector and a comparison between horizontal or vertical pairs of photoreceptors [54–56]. As previously mentioned, the gaze stabilization response of many insects originates with directionally selective wide-field neurons that have a specific orientation in the eye relative to real-world coordinates (e.g. [24,25]). Gaze-stabilizing mechanisms in stomatopods, with their torsionally rotating eyes, would likely require a different architecture that is optimized to a shifting coordinate system. For stomatopods, the apparent direction of motion of a stimulus will depend on the eye's torsional pose. For instance, the direction of a horizontally moving stimulus progressing at a constant angular velocity will appear to change sinusoidally as the eye rotates torsionally (figure 4). Nevertheless, as we have shown, the yaw gaze stabilization performance of a stomatopod eye appears to be independent of both its torsional pose and its rate of torsional rotation. Such a finding suggests that the stomatopod's wide-field motion detection network may be radially symmetrical, which would be novel in any visual system.

Further to this, *O. scyllarus* did not show any evidence of torsional gaze stabilization in response to a torsionally rotating field of view. This is in contrast to many flying insects, such as honeybees [57], wasps [58] and blowflies [59], which exhibit compensatory torsional rotation of the head in order to stabilize their gaze relative to torsional rotation of the visual scene. In insects with halteres, such as dipteran flies, compensatory torsional rotation of the head can be induced either by the visual or haltere systems, while in the hymenopteran insects lacking the mechanosensory information from halteres, torsional head rotations have been shown to be governed purely by the visual system [57–59]. Three-dimensional gaze stabilization is particularly important during flight control as it reduces motion blur and prevents image rotation from degrading optic flow information; translational optic flow being an important visual cue during flight, assisting in flight control, determining self-motion, navigation and landing [57,60,61].

Like many other crustaceans, *O. scyllarus* are benthic, spending the vast majority of their time in contact with the ocean floor or with the walls of their home burrows. Consequently, torsional stability may not be as critical as it would be to a flying insect, at least while the stomatopod is stationary. Several species of benthic crabs have been shown to perform torsional rotation of their eyes in response to vestibular or visual stimuli [62–64]. It is likely that these compensatory eye movements, which have a limited rotational range (less than 15°), act to keep the gaze of the crab stable and level relative to a local horizon during locomotion over a rough terrain [62,63]. However, two substantial differences in the visual systems of stomatopods and crabs limit the comparison between the two animals; stomatopods fixate objects and perform visual scans, while crabs do neither [42,44,46,65].

Although we found no evidence for torsional gaze stabilization in stationary *O. scyllarus* when viewing a torsionally rotating visual scene, it is possible that their repertoire of eye movements may change when they undergo locomotion, either walking or swimming, due to different neural control requirements. Locomotion will induce optic flow in the stomatopod's visual system and, although little is known about their way, they may process and use such information, it is likely to be as useful to stomatopods as to other invertebrates. In addition, while stationary, torsional eye rotations are likely to play a functional role in enhancing certain facets of stomatopod vision: for instance, both *O. scyllarus* and *G. smithii* employ torsional rotations in order to dynamically enhance their linear polarization vision [42]. It has also been hypothesized that torsional rotations may be instrumental in optimally positioning the eyes during scans that are undertaken during the visual inspection of objects [47].

## 5. Conclusion

Although stomatopods display the stereotypical features of optokinetic nystagmus in ocular yaw rotations in response to the horizontal displacement of their visual field, the neural basis of this gaze stabilization system is potentially more complex than that of other crustaceans due to their ability to perform torsional rotations through at least 90° while simultaneously yaw-tracking. Although it is far easier to detect motion in a scene when an eye is stable, the stomatopod's visual system appears to be able to detect and follow translational motion even during torsional rotation. Our findings could be explained by the presence of an unusual radial array of motion detectors in stomatopod visual systems. Such an array has never been described, and it would have to be able to compensate for wide-field translational motion and be unaffected by torsional self-motion of the eyes, at least when the animal is stationary. Such a radial array would allow the stomatopods to detect, and therefore track, the motion of a stimulus in any direction equally across the eye rather than along set directions. A full understanding of this system has biomimetic potential, as a system of motion detection that is insensitive to the negative effects of torsional rotation would have many applications in the realm of machine vision, especially on mobile platforms.

## Supplementary Material

Supplementary Methods (S1)

## Supplementary Material

Supplementary Results (S2)

## Supplementary Material

Supplementary Results II (S3)

## Data accessibility

All data are available through the figshare database: Pitch, yaw and roll during yaw optokinesis: https://figshare.com/s/09470b9fb2a3b2ca94d9. Pitch, yaw and roll during torsional optokinesis: https://figshare.com/s/847b9a5cda4ac0def255.
