# Supplementary material for: Complex gaze stabilization in mantis shrimp
Source: Proc Biol Sci. 2018 May 2;285(1878):20180594. doi: 10.1098/rspb.2018.0594 (PMC5966611; doi:10.1098/rspb.2018.0594)
Supplement: Supplementary Methods (S1) [file rspb20180594supp1.pdf]

Supplementary Material

Materials and Methods

Yaw Optokinesis

Yaw optokinesis was elicited by the horizontal motion of a black and white grating on the inner face of a rotating drum, following the method of Daly et al (2017), and as shown in figure 1*b-d*. Seventeen *O. scyllarus* were placed individually in an artificial 'burrow' (a horizontally orientated, 30 mm diameter 80 mm long plastic tube) in a 200 mm radius cylindrical transparent acrylic (Perspex™) aquarium fixed in the centre of a larger rotating Perspex™ cylinder (diameter 300 mm, height 400 mm). The central cylinder was filled to a depth of 150 mm with seawater from the animal's home aquarium. The external rotating cylinder was not filled with water and was free to spin in the horizontal plane, about the vertical z-axis, in either the clockwise or anticlockwise direction, driven by an electric motor (970D, Como drills, Kent, UK) at an angular speed of 11.50±0.46 $°s^{-1}$ (mean ± standard deviation). The visual stimulus was provided by a grating comprising 24 pairs of black and white stripes of equal widths (19.6mm) printed on A3 paper and attached to the inner side of the drum, with each pair subtending a visual angle of 15° from the position of the experimental animal. The Michelson contrast between the black and white components of the grating was 93.8% in the 420-700 nm range of the spectrum. Each animal was shown six presentations of the drum: three in which the drum rotated in the clockwise direction and three anticlockwise. The three-dimensional rotation of the eyes was recorded using a pair of calibrated stereoscopic video camcorders (Panasonic HC-X900, Osaka, Japan; image resolution 1080p, 50fps) and tracked using MATLAB (version 2015b, Mathworks, Massachusetts, USA) using the method previously described by Daly et al (2017).

The rotation of the drum causes wide-field horizontal motion of the visual field (figure 1*d*), which elicits a stereotypical OKR-type gaze stabilisation response involving yaw rotation of the eye. During optokinesis, an eye tracks the movement of the visual field before performing a rapid counter rotation to a 'reset' its position. The performance of an eye when stabilising its gaze can be quantified using the relative velocity ratio (previously termed 'gain' by Cronin et al (1991)), which is the ratio between the angular velocity of the drum and the angular velocity of the eye . Since the drum is only free to rotate about the z-axis, the angular speed of the drum will be purely in the x−y yaw plane. The relative velocity ratio in the yaw degree of freedom, S_Y, is given by

$$S_Y = \frac{eye\ angular\ velocity}{drum\ angular\ velocity} \quad \text{(eq. S1)}$$

where the angular velocity of the eye is in the yaw (side-to-side) degree of freedom.

If the angular velocity of yaw movements exactly matches that of the drum, $S_Y=1$, corresponding to perfect gaze stabilisation. In reality, due both to the delay caused by the finite response time of the animal visual system and the need for the closed-loop feedback system controlling the eyes' tracking movements, S_Y is usually <1. If $S_Y>1$, the angular speed of the eye exceed that of the drum, and if the eye is stationary, $S_Y=0$. If the eye rotates in the opposite direction to the drum, as it does during the fast reset phase of optokinetic nystagmus, then $S_Y<0$. In some of the following analyses S_Y is calculated only during the smooth tracking phase of the optokinetic nystagmus, in which the eye rotates in the same direction of the drum, such that $S_Y\geq0$. Several figures include values of S_Y during both slow (tracking) and fast (reset) phases to demonstrate the overall trend in yaw rotation of the eyes during optokinesis.

The angular velocity of the eyes used to calculate S_Y is derived from the angular pose in each frame of the video, rather than manually selecting regions of the sawtooth responses. This was to avoid human error in determining when a movement began or ended, and to ensure that all available data was used. Similarly, this ensured that equal numbers of data were used from each eye and each individual animal.

Torsional Optokinesis

A torsionally rotating field of view was created by turning the rotating drum on its side such that its axis of rotation was horizontal (figure 1*e-g*). A narrow slit was cut into the drum to allow for filming from above with only minimal occlusion of the stereoscopic cameras' fields of view. The stomatopod was placed in a cuboid tank that rested on a cantilevered Perspex bridge facing the closed end of the torsional rotating drum. The closed end of the rotating drum was covered with a radial pattern of 24 pairs of black and white stripes (figure 1*g*), with each pair extending over an angle of 15° to intercept the grating pattern on the drum surface. As for the yaw optokinesis experiment, the inner face of the side of the rotating drum was covered with 24 pairs of black and white stripes printed on A3 paper that, from the perspective of the experimental animal, subtended a viewing angle of 15°. The drum was able to rotate both clockwise and anticlockwise. Six *O. scyllarus* were placed individually in the

central stationary aquarium and shown six presentations of drum movement: a 40s duration presentation at three angular speeds (slow: 3.41±0.04 $°s^{-1}$, medium: 7.48±0.42$°s^{-1}$, fast: 12.74±0.16 $°s^{-1}$ (mean ± standard deviation)) of the torsional drum in the clockwise and anticlockwise directions, the order of drum speed and direction being pseudorandomised. The yaw, pitch and torsion angles of the eye during each frame in the real world coordinate system were found using the stereoscopic tracking method outlined previously by Daly et al (2017).

In analogy with the relative velocity ratio for yaw optokinesis, the torsional gaze stabilisation performance was quantified using the torsional relative velocity ratio, S_T, the ratio of the torsional velocity of the drum (rotation in the x-z plane, about a horizontal axis) and the angular velocity of torsional rotation of the eye

$$S_T = \frac{eye\ torsional\ angular\ velocity}{drum\ angular\ velocity} \qquad \text{(eq. S2).}$$

An additional consideration when calculating S_T is the perceived angular torsional rotation of the drum, which will vary with the yaw pose of the eye. For instance, if the eye is facing forwards (θ_Y=0°), directly towards the radial pattern at the closed end of the drum, then the eye will experience the true torsional velocity of the drum. If, however, the eye is rotated in the yaw degree so that it is facing the side of the drum (θ_Y=90°), the eye will no longer experience a torsional rotation of the visual field, but will instead experience the rotation of the drum as in the pitch direction. In order to avoid this complication, the analyses of torsional rotation were restricted to yaw poses of θ_Y<30°.

References

Cronin TW, Marshall NJ, Land MF. (1991) Optokinesis in gonodactyloid mantis shrimps (Crustacea; Stomatopoda; Gonodactylidae). *J. Comp. Physiol. A* **168**, 233–240. (doi:10.1007/BF00218415)

Daly IM, How MJ, Partridge JC, Roberts NW. (2017) The independence of eye movements in a stomatopod crustacean is task dependent. *J. Exp. Biol.* **220**, 1360–1368. (doi:10.1242/jeb.153692)
