# Supplementary material for: Complex gaze stabilization in mantis shrimp
Source: Proc Biol Sci. 2018 May 2;285(1878):20180594. doi: 10.1098/rspb.2018.0594 (PMC5966611; doi:10.1098/rspb.2018.0594)
Supplement: Supplementary Results (S2) [file rspb20180594supp2.pdf]

Supplementary Material

Supplementary Results

The amplitude and frequency of the yaw rotations during yaw optokinesis are highly variable, even within the same animal responding to the same stimulus, as shown in figure S1*a*-*c*. Across both eyes of all seventeen animals, the median angular distance, $\delta\vartheta_Y$, over which the stripes were stabilised during the slow phase of optokinetic nystagmus before the fast reset phase was $\delta\vartheta_Y$=9.42±1.16° (median ± 95% CI), with no significant difference in $\delta\vartheta_Y$ between the two eyes (left eye $\delta\vartheta_Y$=8.54±1.16° (median ± 95% CI), right eye $\delta\vartheta_Y$=10.64±1.20° (median ± 95% CI), GLMM, χ^2=1.39, *n*=17, *p*=0.238). There was a significant difference in the angular distance tracked between the two directions of drum rotation, with a significantly greater angular distance during anticlockwise drum rotation (clockwise $\delta\vartheta_Y$=11.16±1.45° (median ± 95% CI), anticlockwise $\delta\vartheta_Y$=7.88±1.09° (median ± 95% CI), GLMM, χ^2=13.19, *n*=17 *p*<0.001).

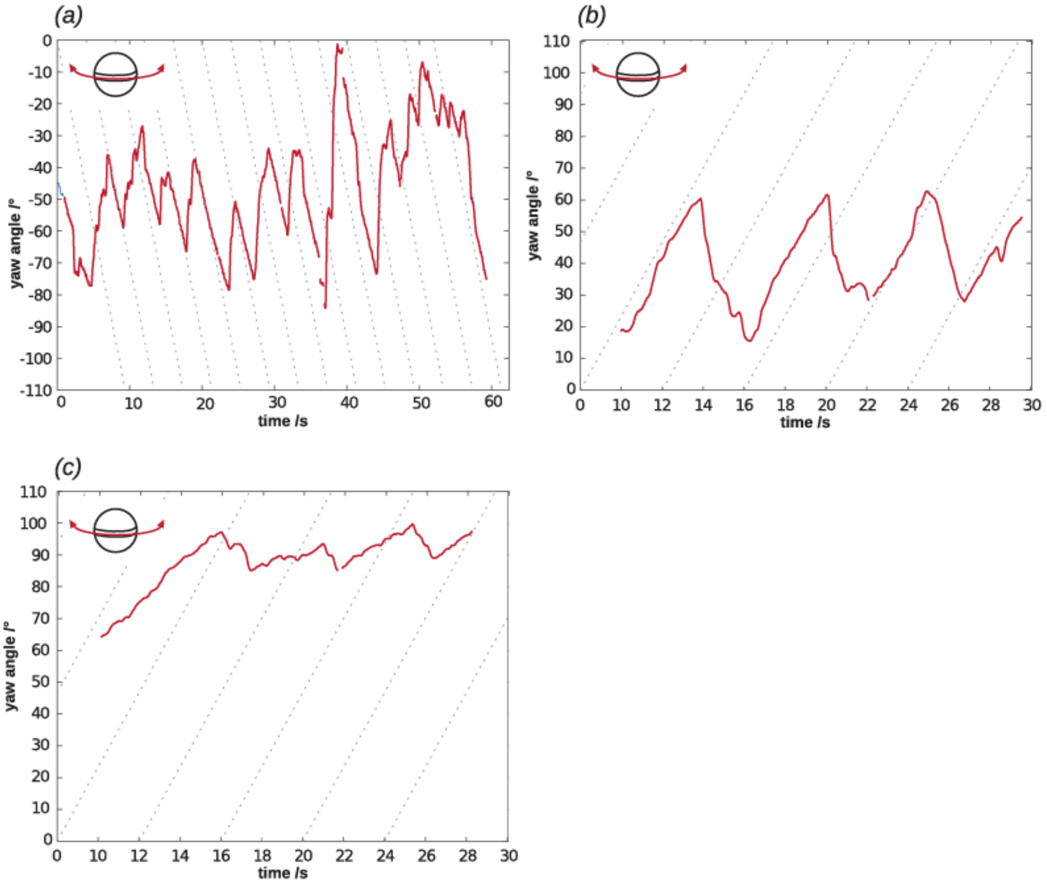

Figure S1: The optokinetic yaw response of the eyes is highly variable, even within the same animal.
