# Supplementary material for: Complex gaze stabilization in mantis shrimp
Source: Proc Biol Sci. 2018 May 2;285(1878):20180594. doi: 10.1098/rspb.2018.0594 (PMC5966611; doi:10.1098/rspb.2018.0594)
Supplement: Supplementary Results II (S3) [file rspb20180594supp3.pdf]

Supplementary Material

Supplementary Results

Table S1.

Degrees of rotational freedom	Maximum cross correlation coefficient (median ± 95% CI)	Wilcoxon-signed rank (V)	*p*-value
yaw-torsion (*left eye*)	0.01±0.20	95	0.381
yaw-torsion (*right eye*)	0.04±0.21	117	0.055
yaw-pitch (*left eye*)	-0.01±0.14	66	0.619
yaw-pitch (*right eye*)	0.02±0.13	95	0.381
torsion-pitch (*left eye*)	0.00±0.17	84	0.723
torsion-pitch (*right eye*)	0.00±0.13	82	0.795

Table S1: *The median (± 95% confidence interval (CI)) maximum cross correlation coefficient between each of the three degrees of rotational degrees of freedom for the left and right eyes and the results of a Wilcoxon sign-ranked test with a median of 0 (n=17).*
